# Breast Tumor Cell-Stimulated Bone Marrow-Derived Mesenchymal Stem Cells Promote the Sprouting Capacity of Endothelial Cells by Promoting VEGF Expression, Mediated in Part through HIF-1α Increase

**DOI:** 10.3390/cancers14194711

**Published:** 2022-09-27

**Authors:** Wootak Kim, Aran Park, Hyun Hee Jang, Seung-Eun Kim, Ki-Sook Park

**Affiliations:** 1 Department of Biomedical Science and Technology, Graduate School, Kyung Hee University, Seoul 02447, Korea; 2 Graduate School of Biotechnology, Kyung Hee University, Yongin 17104, Korea; 3 Department of Genetics and Biotechnology, Kyung Hee University, Yongin 17104, Korea; 4 East-West Medical Research Institute, Kyung Hee University, Seoul 02447, Korea

**Keywords:** bone marrow-derived mesenchymal stem cells, breast tumor, VEGF, HIF-1α, ROS, JAK/Stat

## Abstract

**Simple Summary:**

ROS and JAK/Stat3 cooperatively upregulate the expression of HIF-1α in bone marrow-derived mesenchymal stem cells under normoxic conditions in response to breast tumor cells. The upregulation of HIF-1α contributes in part to the increase in VEGF expression in the bone marrow-derived mesenchymal stem cells. Bone marrow-derived mesenchymal stem cells improve the angiogenic sprouting capacity of mature endothelial cells in a VEGF-dependent manner.

**Abstract:**

Breast tumor cells recruit bone marrow-derived mesenchymal stem cells (BM-MSCs) and alter their cellular characteristics to establish a tumor microenvironment. BM-MSCs enhance tumor angiogenesis through various mechanisms. We investigated the mechanisms by which BM-MSCs promote angiogenesis in response to breast tumor. Conditioned media from MDA-MB-231 (MDA CM) and MCF7 (MCF7 CM) breast tumor cells were used to mimic breast tumor conditions. An in vitro spheroid sprouting assay using human umbilical vein endothelial cells (HUVECs) was conducted to assess the angiogenesis-stimulating potential of BM-MSCs in response to breast tumors. The ROS inhibitor N-acetylcysteine (NAC) and JAK inhibitor ruxolitinib attenuated increased HIF-1α in BM-MSCs in response to MDA CM and MCF7 CM. HIF-1α knockdown or HIF-1β only partially downregulated VEGF expression and, therefore, the sprouting capacity of HUVECs in response to conditioned media from BM-MSCs treated with MDA CM or MCF7 CM. Inactivation of the VEGF receptor using sorafenib completely inhibited the HUVECs’ sprouting. Our results suggest that increased HIF-1α expression under normoxia in BM-MSCs in response to breast tumor cells is mediated by ROS and JAK/Stat3, and that both HIF-1α-dependent and -independent mechanisms increase VEGF expression in BM-MSCs to promote the angiogenic sprouting capacity of endothelial cells in a VEGF-dependent manner.

## 1. Introduction

Bone marrow-derived mesenchymal stem cells (BM-MSCs) migrate into injured tissues and contribute to tissue regeneration [1,2,3]. The migrated BM-MSCs differentiate into various types of somatic cells, including endothelial cells and pericytes [3,4], and secrete anti-inflammatory cytokines, growth factors, and proangiogenic factors such as vascular endothelial growth factor (VEGF) and basic fibroblast growth factor [2,5,6].

BM-MSCs have tumor-tropic activity as well as regenerative activity. Angiogenesis mediates the formation of new blood vessels from the preexisting vessels and generates tumor vasculature that is required for tumor growth and metastasis [7]. BM-MSCs are actively recruited to tumors, as to the injured tissues, and enhance tumor angiogenesis, tumor growth, and metastasis [8,9,10,11].

BM-MSCs dramatically accelerate angiogenesis through various mechanisms. BM-MSCs have been shown to differentiate into tumor endothelial cells [12]. BM-MSCs are integrated into tumor blood vessels and function as cells expressing pericyte markers, such as α-smooth muscle actin [13]. Moreover, BM-MSCs secrete interleukin 6, which stimulates cancer cells to induce the mobilization of tumor endothelial cells and tumor vessel formation [14]. BM-MSCs upregulate tumor angiogenesis by secreting stromal-cell-derived factor 1, which recruits endothelial progenitor cells into the tumor [15]. BM-MSCs have also been shown to secrete proangiogenic factors—including VEGF—to activate tumor endothelial cells and stimulate tumor angiogenesis [16,17].

Hypoxia-inducible transcription factors (HIFs) regulate angiogenesis by enhancing VEGF expression in hypoxia-exposed cells by binding the VEGF promoter [18,19]. HIFs are heterodimers composed of an α subunit (HIF-1α, HIF-2α, or HIF-3α) and β subunit (HIF-1β) [19,20]. The expression of HIF α proteins is regulated by oxygen concentration, but HIF β proteins are constitutively expressed regardless of oxygen concentration [19,20]. HIF-1α is targeted to proteasome-dependent degradation in response to normoxia [21], wherein it undergoes ubiquitylation mediated by the von Hippel–Lindau tumor suppressor E3 ligase complex following hydroxylation mediated by prolyl hydroxylases, known as prolyl hydroxylase domain (PHD) proteins, which show oxygen-dependent enzyme activity [21,22]. However, it has also been reported that HIF-1α may be stable even under normoxia [23]. Pyruvate, lactate, and oncometabolites such as succinate and fumarate can stabilize the HIF-1α protein [23,24,25]. Reactive oxygen species (ROS) have been also shown to regulate the activity and stability of HIF-1α under normoxia [23,26]. The transcription of HIF-1α can be stimulated by the Janus kinase (JAK)/signal transducer and activator of transcription (Stat) signaling pathway and pro-survival signaling pathways such as the ERK and Akt pathways [23,27,28].

The expression of VEGF—an essential mediator of angiogenesis—has been found to be regulated both dependent and independent of HIF-1α. Peroxisome-proliferator-activated receptor-γ coactivator-1α—the transcriptional coactivator that is induced by low oxygen—induces the expression of VEGF independently of HIF-1α [29]. VEGF has been shown to be upregulated via various mechanisms, such as acidosis, oncogenic activation, elevated ROS, and transforming growth factor-beta (TGF-β) levels, and activated through the JAK/Stat signaling pathway, independent of oxygen concentration [30,31,32,33,34,35,36,37].

The JAK/Stat signaling pathway controls angiogenesis through the regulation of VEGF expression. There are four mammalian JAKs (JAK1, JAK2, JAK3, and TYK2) and seven mammalian Stats (Stat1, Stat2, Stat3, Stat4, Stat5A, Stat5B, and Stat6) [38,39]. JAKs are the intracellular non-receptor tyrosine kinases that are activated in response to cytokines and hormones binding to their receptors [38,39]. Activated JAKs phosphorylate Stats which, in turn, enter the nucleus to regulate the transcription of target genes [38,39].

BM-MSCs highly express VEGF under physiological and pathophysiological conditions. For example, BM-MSCs enhanced angiogenesis in the myocardium of a myocardial infarction animal model via HIF-1α-induced VEGF secretion [40]. BM-MSCs enhanced HIF-1α-dependent VEGF expression in response to the inflammatory tumor microenvironment to promote angiogenesis in colon and prostate cancers [17,41]. It has been shown that BM-MSCs also enhance vascularity in breast tumors [42], but little is known about the mechanisms mediating the increase in angiogenesis of breast tumors. In this study, we investigated whether BM-MSCs, in response to breast tumor cells, promote the sprouting capacity of human umbilical vein endothelial cells (HUVECs) in a VEGF-dependent manner. We also investigated whether the high VEGF expression in BM-MSCs is dependent on or independent of HIF-1α, as well as the mechanisms that increase HIF-1α expression in BM-MSCs in response to breast tumor cells.

## 2. Materials and Methods

### 2.1. Cell Culture

Human BM-MSCs were obtained from Lonza (Basel, Switzerland) and were maintained with Mesenchymal Stem Cell Growth Medium (Lonza). The cells between passages 4 and 7 were used for all experiments. MDA-MB-231 cells and MCF7 cells were obtained from the American Type Culture Collection (ATCC, Manassas, VA, USA). MDA-MB-231 cells were cultured in Dulbecco’s modified Eagle medium/high glucose (DMEM/HG; HyClone, Logan, UT, USA), supplemented with 10% heat-inactivated fetal bovine serum (FBS; Thermo Fisher Scientific, Waltham, MA, USA) and 100 U/mL penicillin/streptomycin (P/S; Thermo Fisher Scientific). MCF7 cells were cultured in DMEM/F-12 (1:1) (Thermo Fisher Scientific) supplemented with 10% FBS, 2 mM L-glutamine (Thermo Fisher Scientific) and 100 U/mL P/S. Human umbilical vein endothelial cells (HUVECs; Lonza) were maintained on 0.2% gelatin from a bovine skin (Sigma-Aldrich, St. Louis, MO, USA)-coated dish using Endothelial Cell Growth Media-Plus (Lonza), and HUVECs between passages 4 and 8 were used for all experiments. All of the cells were maintained at 37 °C in a humidified incubator containing 5% CO_2_.

### 2.2. Conditioned Media Preparation

#### 2.2.1. Conditioned Media of Breast Tumor Cells

MDA-MB-231 and MCF7 cells were cultured using their culture media until confluence. The cells were then washed with phosphate-buffered saline (PBS) and incubated for 3 days with DMEM/HG supplemented with 100 U/mL P/S or DMEM/F-12 (1:1) supplemented with 2 mM L-glutamine and 100 U/mL P/S for MDA-MB-231 or MCF7 cells, respectively. To prepare conditioned medium (CM) for the control, DMEM/HG supplemented with 100 U/mL P/S or DMEM/F-12 (1:1) supplemented with 2 mM L-glutamine and 100 U/mL P/S was incubated for 3 days under cell-free conditions. The CM from MDA-MB-231 (MDA CM), MCF7 (MCF7 CM), or the control (CON CM) was filtered through a 0.2 μm filter (Corning, Cornyn, NY, USA), aliquoted, and stored at −80 °C until use.

#### 2.2.2. Conditioned Media from BM-MSCs Primed with MDA CM or MCF7 CM

BM-MSCs were cultured in 6-well plates until 80–90% confluence and incubated for 24 h with DMEM/low glucose (DMEM/LG) supplemented with 2 mM L-glutamine and 100 U/mL P/S. Then, the BM-MSCs were rinsed with PBS and incubated with MDA CM, MCF7 CM, or CON CM for 48 h. The CM from the cells was collected, filtered through a 0.2 μm filter, aliquoted, and stored at −80 °C until use. For the control, MDA CM, MCF7 CM, or CON CM was incubated under cell-free conditions for 48 h. For knockdown experiments, BM-MSCs were transfected with siRNA targeting *HIF1A* or *ARNT* and incubated for 24 h prior to adding MDA CM or CON CM.

### 2.3. Western Blot Analysis

BM-MSCs were cultured until to 80–90% confluence and serum-starved overnight using DMEM/LG supplemented with 2 mM L-glutamine and 100 U/mL P/S. Then, the BM-MSCs were treated with MDA CM, MCF7 CM, or CON CM for the indicated times. For JAK and ROS inhibition, the cells were pretreated with ruxolitinib (Cayman Chemical, Ann Arbor, MI, USA) and N-acetylcysteine (NAC; Sigma-Aldrich), respectively, for 30 min prior to the CM treatment. BM-MSCs were rinsed twice with ice-cold PBS and lysed using 2× SDS buffer (100 mM Tris-HCl, pH 6.8, 20% (*v*/*v*) glycerol, 2% (*v*/*v*) sodium dodecyl sulfate (SDS), 0.001% (*w*/*v*) bromophenol blue, and 10% (*v*/*v*) β-mercaptoethanol) at 25 °C for 5 min. The cell lysates were collected by scrapping and denatured by heating at 95 °C for 5 min. Western blot analysis was performed with the following primary antibodies: anti-HIF-1α (1:800-1:1000; Cell Signaling Technology, Danvers, MA, USA), anti-phospho-Stat3 (pStat3; 1:500–1:800; Cell Signaling Technology), anti-Stat3 (1:1000; Cell Signaling Technology), and α-tubulin (1:30000–1:50000, Sigma-Aldrich). Densitometry of the bands obtained was performed using the ImageJ software (NIH, Bethesda, MD, USA).

### 2.4. Quantitative Real-Time Polymerase Chain Reaction (qRT-PCR)

Total RNA was isolated using the TRIzol reagent (Invitrogen, Waltham, MA, USA), and cDNA was synthesized using SuperScript III Reverse Transcriptase (Invitrogen), according to the manufacturers’ protocols. Quantitative reverse-transcriptase polymerase chain reaction (qRT-PCR) was performed using the SYBR Green reagent (Invitrogen). The human ribosomal protein S9 gene (RPS9) was used as an endogenous control. The primer sequences were as follows: *HIF1A* (forward): 5′-TTTGGCAGCAACGACACAGA-3′; *HIF1A* (reverse): 5′-CGTTTCAGCGGTGGGTAATG-3′; *ARNT* (forward): 5′-CCACAGGAACTCTTAGGAA-3′, *ARNT* (reverse): 5′-CATGACAGACAGCACTTG-3′; VEGFA (forward): 5′-CTGCTCTACCTCCACCATGC-3′, VEGFA (reverse): 5′-AGCTGCGCTGATAGACATCC-3′; SLC2A1 (forward): 5′-CTTTGTGGCCTTCTTTGAAGT-3′, SLC2A1 (reverse): 5′-CCACACAGTTGCTCCACAT-3′; RPS9 (forward): 5′-CTGACGCTTGATGAGAAGGAC-3′, RPS9 (reverse): 5′-CAGCTTCATCTTGCCCTCAT-3′.

### 2.5. Immunocytochemistry

BM-MSCs were cultured until 80–90% confluence on type I collagen (Nitta Gelatin, Yao, Osaka, Japan)-coated cover glasses in 24-well plates and serum-starved overnight using DMEM/LG supplemented with 2 mM L-glutamine and 100 U/mL P/S. Then, BM-MSCs were treated with MDA CM for 6 h. The cells were rinsed with ice-cold PBS and fixed with 4% formaldehyde (Sigma-Aldrich) for 10 min on ice. After fixation, immunocytochemistry was performed using antibodies against HIF-1α (1:100; Cell Signaling Technology) and Alexa-Fluor-488-labeled goat anti-mouse IgG antibody (1:500; Invitrogen) according to standard protocols [43]. Nuclei were stained using 4,6-diamidino-2-phenylindole (DAPI; Invitrogen) for 10 min, and actin was stained using Alexa-Fluor-546-tagged phalloidin (1:1500; Invitrogen). The cells were imaged using a Zeiss LSM 700 confocal microscope (Carl Zeiss, Oberkochen, Germany).

### 2.6. Transfection of Cells with Small Interfering RNAs (siRNA)

BM-MSCs were transfected with siRNA using Lipofectamine RNAiMAX (Invitrogen) following the manufacturer’s instructions. Scrambled siRNA (OriGene, Rockville, MD, USA) was used as a negative control. siRNAs were synthesized by Genolution (Seoul, South Korea) and their sequences were as follows: HIF-1α sense: 5′-GUGGUUGGAUCUAACACUAUU-3′, HIF-1α antisense: 5′-UAGUGUUAGAUCCAACCACUU-3′; HIF-1β #1 sense: 5′-GGUCAGCAGUCUUCCAUGAUU-3′, HIF-1β #1 antisense: 5′-UCAUGGAAGACUGCUGACCUU-3′; HIF-1β #2 sense: 5′-CCAUCUUACGCAUGGCAGUUUUU-3′, HIF-1β #2 antisense: 5′- AAACUGCCAUGCGUAAGAUGGUU-3′. Mixtures with equal amounts of HIF-1β siRNAs #1 and #2 were used to knock down HIF-1β.

### 2.7. In Vitro Spheroid Sprouting Assay Using HUVEC Spheroids

HUVECs were suspended at a density of 1.33 × 10^4^ cells/mL in Medium 199 (Sigma-Aldrich) supplemented with 10% (*v*/*v*) FBS and 0.2% (*v*/*v*) methylcellulose (Sigma-Aldrich). The cell suspension (30 µL) was seeded into the non-adherent lid of a Petri dish. The lid was turned upside-down and incubated for 24 h to assemble single spheroids (~400 cells/spheroid). The HUVEC spheroids were collected in PBS and centrifuged at 200× *g* for 5 min. The spheroids were then resuspended in a methylcellulose mixture containing 20% (*v*/*v*) FBS and 0.95% (*v*/*v*) methylcellulose in Medium 199. For preparing the type I collagen gel mixture, 3 mg/mL type I-A collagen (Nitta Gelatin) was mixed with 10× Medium 199 (Sigma-Aldrich) and 10× reconstitution buffer (0.05 N NaOH, 0.261 M NaHCO_3_, and 0.2 M HEPES) at a ratio of 8:1:1 on ice. The collagen mixture was then added to the methylcellulose mixture containing spheroids at a 1:1 ratio on ice. This mixture (0.7 mL) was transferred into a 24-well plate and immediately incubated at 37 °C and 5% CO_2_ for 30 min to polymerize the gel. Then, 100 μL of Medium 199 containing 20 ng/mL VEGF_165_ (PeproTech, Rocky Hill, NJ, USA) or CM from BM-MSCs primed with MDA CM, MCF7 CM, or CON CM was added on top of the collagen gel containing HUVEC spheroids, and the mixture was incubated for 24 h to induce spheroid sprouting. If necessary, 5 µM sorafenib (Sigma-Aldrich) was pretreated for 30 min prior to the treatment with VEGF_165_ or the CM. At least 10 spheroids were analyzed per group, and both the number of sprouts and the sprout length of all sprouts per spheroid (i.e., cumulative sprout length) were measured using the ImageJ software (NIH).

### 2.8. Statistical Analysis

Quantitative data are expressed as the mean ± SD. Statistical analysis was performed using Student’s *t*-test in GraphPad version 9.4.0 (GraphPad Software Inc.). A value of *p* < 0.05 was considered significant.

## 3. Results

### 3.1. BM-MSCs Show Increased HIF-1α Expression in Response to Breast Tumor-Mimicking Conditions

**Figure 1:**
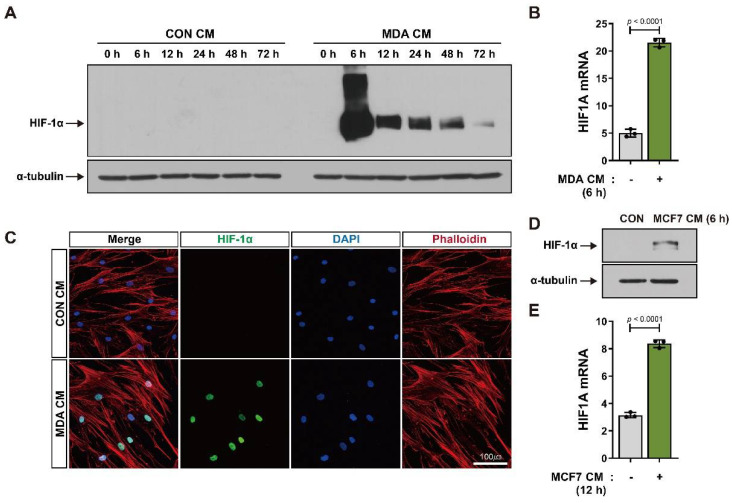
Increase in HIF-1α expression in BM-MSCs in response to breast tumor-mimicking conditions: (**A**,**B**) Protein and mRNA levels of HIF-1α in BM-MSCs treated with the control conditioned medium (CON CM) or conditioned medium from MDA-MB-231 cells (MDA CM) for the indicated times; α-tubulin was used as an internal control for Western blot analysis. (**C**) Immunocytochemistry of BM-MSCs treated with CON CM or MDA CM for 6 h; actin was stained red with phalloidin and nuclei were stained blue with DAPI. (**D**,**E**) Protein and mRNA levels of HIF-1α in BM-MSCs treated with CON CM or conditioned medium from MCF7 (MCF7 CM) cells for 6 h and 12 h, respectively. Results are presented as the mean ± SD. Uncropped Western blot images are available in Figure S1.

BM-MSCs are recruited into tumors, where they stimulate angiogenesis and metastasis [9,10]. BM-MSCs were cultured in CM from breast tumor cells (MDA-MB-231 and MCF7) that simulate breast tumor conditions, and their HIF-1α expression was investigated. HIF-1α levels were higher in BM-MSCs treated with CM from the breast tumor cell line MDA-MB-231 (MDA CM) than with CM from the control (CON CM). HIF-1α levels dramatically increased after 6 h of MDA CM treatment and gradually reduced by 48 h (Figure 1A). Furthermore, in BM-MSCs treated with MDA CM, HIF-1α mRNA levels were upregulated (Figure 1B), and the HIF-1α protein that functions as a transcription factor was localized in the nucleus (Figure 1C). Conditioned media from another breast cancer cell line—MCF7 (MCF7 CM)—also induced an increase in the expression of both HIF-1α protein and HIF-1α mRNA in BM-MSCs (Figure 1D,E).

### 3.2. ROS and JAK/Stat3 Signaling Mediate HIF-1α Induction in BM-MSCs in Response to Breast Tumor-Mimicking Conditions

**Figure 2:**
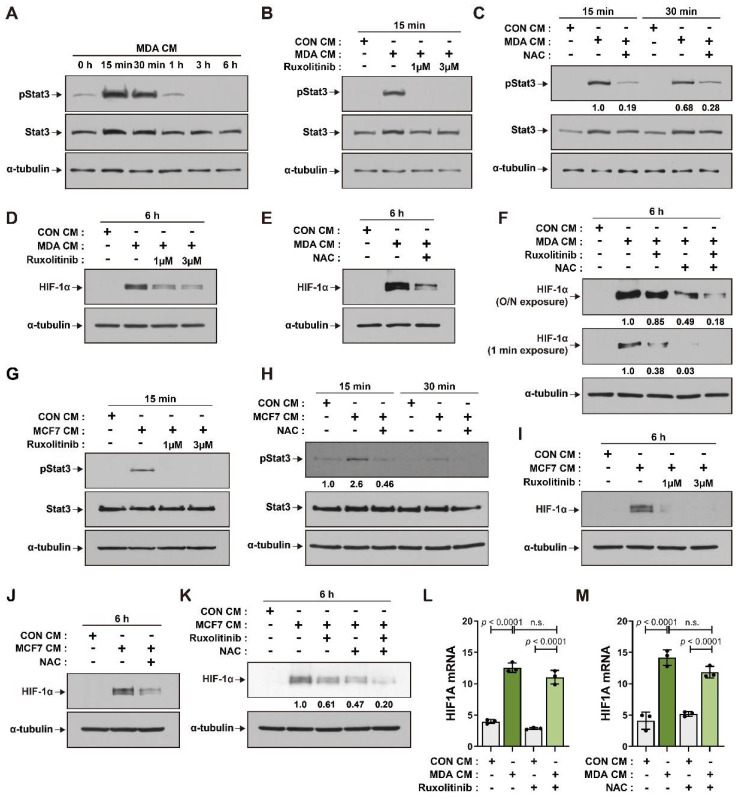
Increase in HIF-1α protein levels in BM-MSCs in response to breast tumor-mimicking conditions via JAK/Stat3 signaling and ROS: (**A**–**K**) Western blot analysis of phosphorylated Stat3 (pStat3), Stat3, and HIF-1α in BM-MSCs treated with CON CM, MDA CM, and MCF7 CM, and in BM-MSCs pretreated with ruxolitinib (1 μM or 3 μM) and/or NAC (5 mM) followed by treatment with CON CM, MDA CM, or MCF7 CM for the indicated times; α-tubulin was used as an internal control; O/N exposure = overnight exposure; the numbers under the blot represent the fold value of pStat3 normalized by Stat3 (**C**,**H**) and of HIF-1α normalized by α-tubulin (**F**,**K**). (**L**,**M**) qRT-PCR analysis of *HIF1A* mRNA expression in BM-MSCs pretreated with ruxolitinib (3 μM) and/or NAC (5 mM), followed by treatment with CON CM or MDA CM for 12 h. Results are presented as the mean ± SD. n.s.: not significant. Uncropped Western blot images are available in Figure S2.

The activation of JAK/Stat signaling is associated with the establishment of the tumor microenvironment [44,45], and Stats play important roles in regulating the stability of HIF-1α proteins [36,46]. We found that the level of phosphorylated Stat3 increased in BM-MSCs incubated with MDA CM. Stat3 phosphorylation increased at 15 min after MDA CM treatment and declined thereafter (Figure 2A). Ruxolitinib, a selective inhibitor of JAK [47], suppressed the increase in Stat3 phosphorylation in BM-MSCs treated with MDA CM (Figure 2B). ROS induces the activation of JAK/Stat signaling [48] and has also been found to regulate the activity and stability of the HIF-1α protein [26,49,50]. N-acetylcysteine (NAC), an ROS inhibitor, suppressed the phosphorylation of Stat3 in BM-MSCs treated with MDA CM (Figure 2C). Ruxolitinib and NAC suppressed the increase in HIF-1α expression in BM-MSCs treated with MDA CM (Figure 2D,E). The elevated levels of HIF-1α protein in BM-MSCs incubated with MDA CM were more significantly suppressed with ruxolitinib and NAC co-treatment than with ruxolitinib or NAC treatment alone (Figure 2F). BM-MSCs incubated with MCF7 CM showed increased JAK-mediated phosphorylation of Stat3, and NAC inhibited Stat3 phosphorylation in BM-MSCs (Figure 2G,H). The MCF7 CM-mediated increase in HIF-1α was suppressed in ruxolitinib- or NAC-pretreated BM-MSCs (Figure 2I,J), and co-treatment with ruxolitinib and NAC showed an addictive effect and greatly suppressed MCF7 CM-mediated HIF-1α expression (Figure 2K). Stat3 and ROS signaling have been shown to mediate the increase in HIF-1α transcription in both hypoxic and normoxic conditions [27,28,51]. Neither ruxolitinib nor NAC treatment was able to suppress the increase in HIF-1α mRNA levels in BM-MSCs treated with MDA CM (Figure 2L,M).

### 3.3. BM-MSCs Regulate VEGF Expression in Response to Breast Tumor-Mimicking Conditions in Both an HIF-1α-Dependent and HIF-1α-Independent Manner

**Figure 3:**
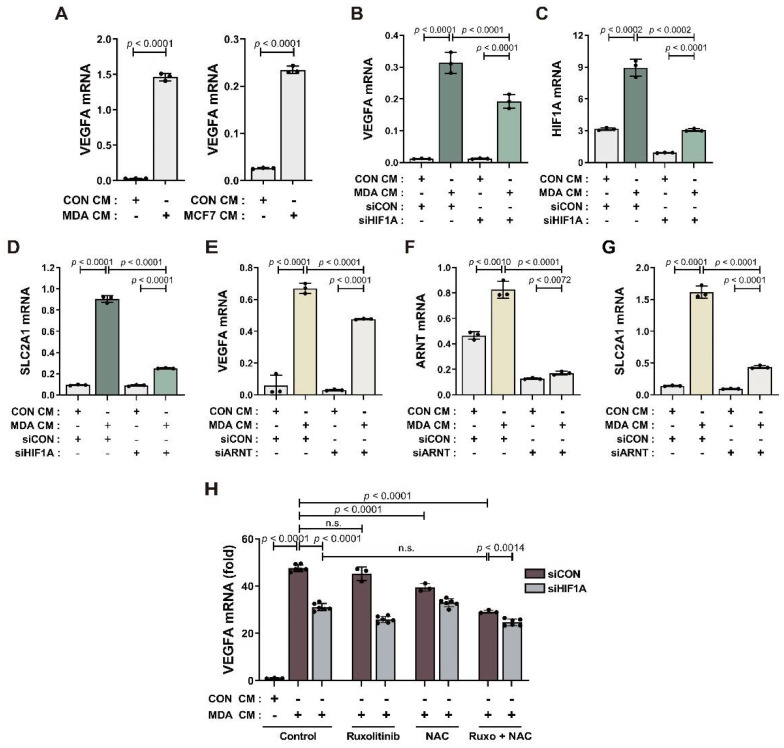
Partial suppression of VEGF expression in response to HIF-1α knockdown: (**A**) qRT-PCR analysis of VEGFA mRNA in BM-MSCs treated with CON CM, MDA CM, or MCF7 CM for 12 h. (**B**–**D**) The expression of VEGF, HIF-1α, and glucose transporter 1 in BM-MSCs transfected with *HIF1A* siRNA or control siRNA prior to treatment with CON CM or MDA CM for 12 h. (**E**–**G**) The expression of VEGF, HIF-1β, and glucose transporter 1 in BM-MSCs transfected with *ARNT* siRNA or control siRNA prior to treatment with CON CM or MDA CM for 12 h. (**H**) qRT-PCR analysis of VEGFA mRNA in BM-MSCs transfected with *HIF1A* siRNA or control siRNA prior to treatment with CON CM or MDA CM for 12 h, and BM-MSCs pretreated with ruxolitinib (1 μM) and/or NAC (5 mM) followed by treatment with CON CM or MDA CM. Results are presented as the mean ± SD.

We previously reported that BM-MSCs show increased VEGF expression in response to MDA CM [52]. In this study, we found that BM-MSCs showed increased VEGF expression in response to both MCF7 CM and MDA CM (Figure 3A). We assessed whether this increase in VEGF transcription was induced by HIF-1α. HIF-1α knockdown by transfection of *HIF1A*-targeted siRNA partially suppressed the increase in the mRNA expression of both HIF-1α and VEGF in BM-MSCs treated with MDA CM, compared to the control siRNA transfection (Figure 3B,C). To exclude the possibility that the partial inhibition of VEGF was due to partial knockdown of HIF-1α, we observed the knockdown effect of HIF-1α on other genes that are upregulated by HIF-1α. Glucose transporter 1 (SLC2A1) is one of the major genes upregulated by HIF-1α. SLC2A1 expression was upregulated in BM-MSCs by MDA CM treatment. The expression of SLC2A1 decreased in HIF-1α-knockdown cells to almost the basal levels found in the control group (Figure 3D), indicating successful knockdown. HIF-1α knockdown was confirmed by performing Western blot analysis (Figure S3). HIF-1β is required for HIF-1α-dependent transcriptional regulation of HIF-1α target genes such as VEGF. HIF-1β knockdown partially suppressed the increase in VEGF expression but almost completely suppressed the increase in SLC2A1 in BM-MSCs treated with MDA CM (Figure 3E–G). These results suggest that ROS and the activity of JAK/Stat3 mediate the increase in HIF-1α of BM-MSCs in response to breast tumor-mimicking conditions (Figure 2). Therefore, we assessed the effects of NAC and the inhibition of JAK/Stat3 signaling on VEGF expression in BM-MSCs treated with MDA CM. Ruxolitinib and NAC suppressed the MDA CM-induced increase in VEGF expression in a synergistic manner, but not completely (Figure 3H). Ruxolitinib and NAC co-treatment partially suppressed VEGF expression, as did *HIF1A* siRNA (Figure 3H).

### 3.4. BM-MSCs Primed with Breast Tumor-Mimicking Conditions Enhance In Vitro Angiogenic Sprouting of HUVECs through VEGF Signaling

**Figure 4:**
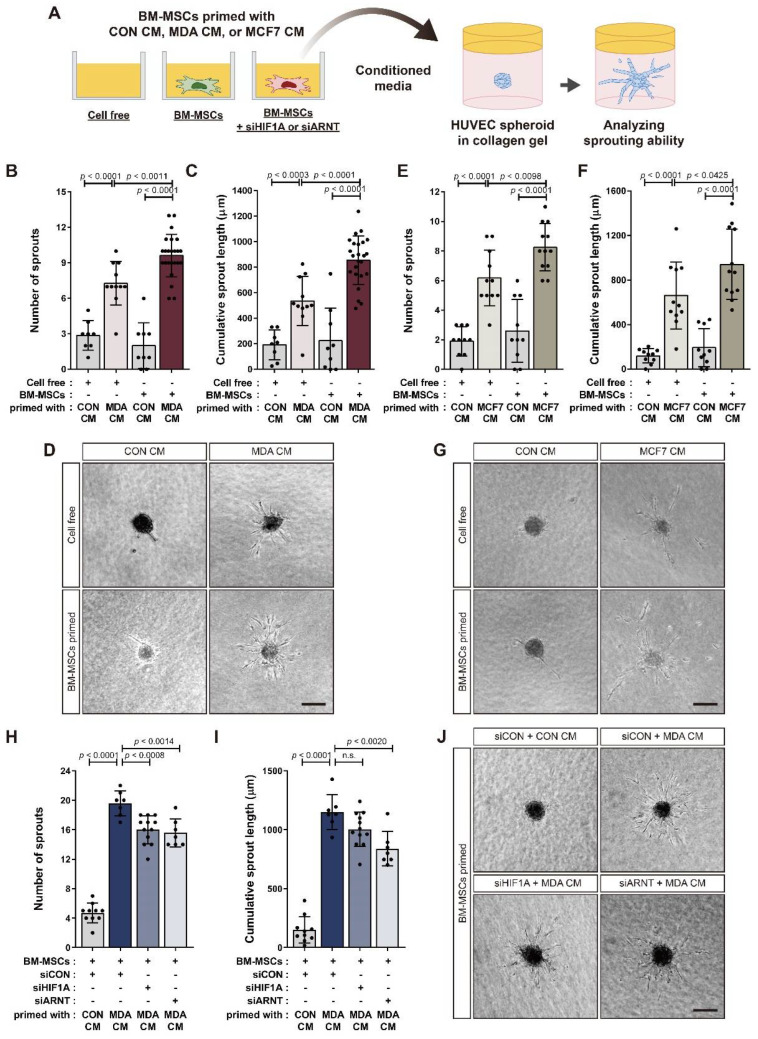
Increase in the angiogenic sprouting capacity of HUVEC spheroids in response to BM-MSCs primed with MDA CM or MCF7 CM: (**A**) Schematic representation of HUVEC spheroid-based sprouting angiogenesis assay. (**B**–**G**) Sprouting capacity of HUVEC spheroids incubated with conditioned media from BM-MSCs primed with CON CM, MDA CM, or MCF7 CM. (**H**–**J**) Sprouting capacity of HUVEC spheroids incubated with conditioned media from HIF-1α- or HIF-1β-knockdown BM-MSCs primed with CON CM or MDA CM. The number of sprouts per spheroid (**B**,**E**,**H**) and the total length of all sprouts originating from a single spheroid (**C**,**F**,**I**) were measured to quantify sprouting capacity. Representative images of each experimental condition are shown (**D**,**G**,**J**). Results are presented as the mean ± SD. Scale bar = 100 μm. n.s.: not significant.

**Figure 5:**
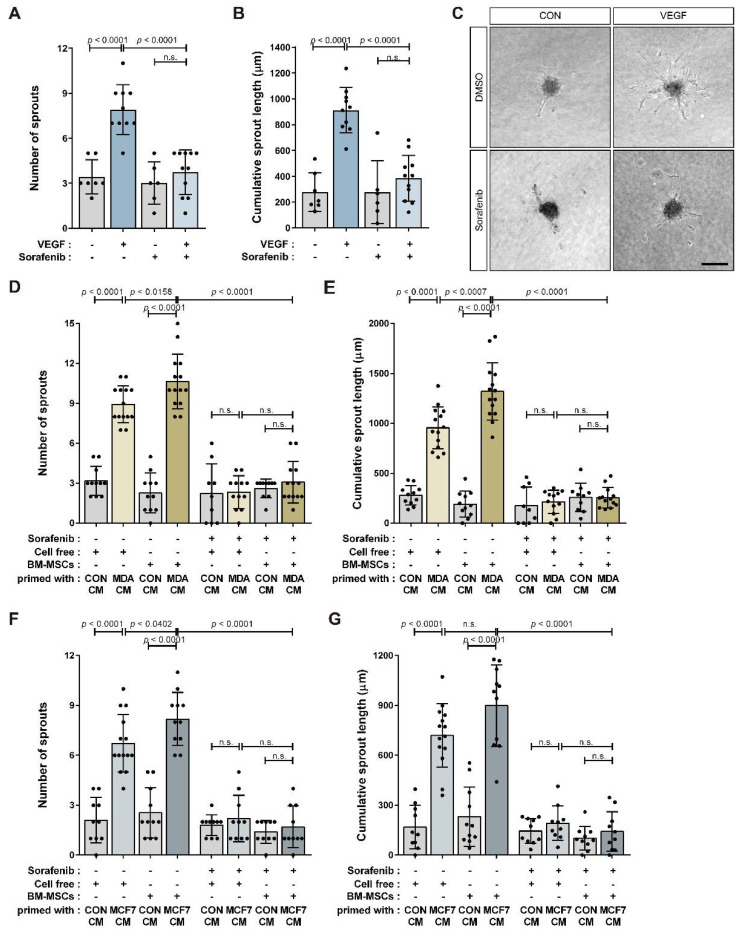
VEGF signaling-mediated promotion of angiogenic activity in HUVECs incubated with conditioned media from BM-MSCs primed with breast tumor-mimicking conditions: (**A**–**C**) VEGF increased the sprouting of HUVEC spheroids, while sorafenib (5 µM) pretreatment inhibited the increase; representative images of each experimental condition (**C**). (**D**–**G**) Sorafenib pretreatment suppressed the sprouting capacity of HUVEC spheroids that increased in response to conditioned media from BM-MSCs primed with CON CM, MDA CM, or MCF7 CM. Results are presented as the mean ± SD. Scale bar = 100 μm.

We analyzed the angiogenic sprouting ability of HUVEC spheroids in response to BM-MSCs primed with MDA CM, MCF7 CM, or CON CM (Figure 4A). The number of sprouts per HUVEC spheroid and the cumulative sprout length of all sprouts per spheroid increased in response to BM-MSCs primed with MDA CM and MCF7 CM compared to CON CM (Figure 4B–G). These results suggest that BM-MSCs treated with MDA CM had increased VEGF expression compared to those treated with CON CM in a partially HIF-1α-dependent manner (Figure 3). Consequently, HIF-1α or HIF-1β knockdown only partially impaired the sprouting capacity of HUVECs in response to BM-MSCs primed with MDA CM (Figure 4H–J). BM-MSCs treated with MDA CM increased VEGF expression by approximately 30-fold compared to BM-MSCs treated with CON CM (Figure S4). We examined whether HUVEC sprouting was mediated by VEGF in BM-MSCs. VEGF treatment promoted angiogenic sprouting in HUVEC spheroids while sorafenib—an inhibitor of various receptor tyrosine kinases, including VEGFR [53,54]—suppressed it (Figure 5A–C). Sorafenib remarkably downregulated angiogenic sprouting in HUVECs that improved in response to the CM from BM-MSCs treated with MDA CM or MCF7 CM (Figure 5D–G).

## 4. Discussion

Tumor angiogenesis is one of the most important tumor-tropic mechanisms. BM-MSCs have been found to increase tumor angiogenesis through various mechanisms, such as secretion of the angiogenic factor VEGF [8,14,16]. In this study, we demonstrated that BM-MSCs enhance the in vitro angiogenic sprouting capacity of HUVECs under normoxic conditions in response to stimulation by breast tumor cells in a VEGF-dependent manner. ROS and JAK/Stat3 cooperatively increased HIF-1α expression under normoxia in BM-MSCs in response to factors secreted from breast tumor cells. This, in turn, increased HIF-1α, and HIF-1α-independent mechanisms increased VEGF expression in BM-MSCs.

The hyperactivation of Stats that occurs in tumor cells and non-transformed cells inside tumors is associated with metastasis, immunosuppression, proliferation, and angiogenesis [44,45]. Stats regulate HIF-1α stability [36,46]. ROS that activate JAK/Stat signaling [48] enhance the stability of HIF-1α through various mechanisms, such as by modulating PHD enzyme activity [49]. The ROS inhibitor NAC and the JAK inhibitor ruxolitinib suppressed the increase in HIF-1α protein in BM-MSCs in response to breast tumor-mimicking conditions. NAC and ruxolitinib suppressed the phosphorylation of Stat3 in BM-MSCs treated with MDA CM or MCF7 CM. These results suggest that ROS function upstream of Stat3 to enhance HIF-1α expression in BM-MSCs in response to breast tumor-mimicking conditions. However, the mechanism by which ROS activates Stat3 in BM-MSCs in response to breast tumor-mimicking conditions and the mechanism by which ROS and JAK/Stat3 increase HIF-1α expression remain unclear and must be further investigated. The levels of both HIF-1α mRNA and HIF-1α protein increased in BM-MSCs in response to breast tumor-mimicking conditions. It has been reported that ROS activate the *HIF1A* promoter via nuclear factor kappa B (NF-κB) or NRF2 [51,55]. However, neither ROS nor JAK/Stat3 was associated with the increase in HIF-1α mRNA in BM-MSCs treated with MDA CM. Whether the increase in HIF-1α mRNA is due to increased transcription of HIF-1α in BM-MSCs in response to breast tumor-mimicking conditions or due to HIF-1α mRNA stabilization is not yet known. Furthermore, it is unclear whether the increase in HIF-1α mRNA contributes to the increase in HIF-1α protein in BM-MSCs faced with breast tumors. Therefore, further experiments are required to clarify the mechanisms that increase HIF-1α mRNA levels in BM-MSCs and to determine whether the increase in HIF-1α mRNA contributes to the increase in HIF-1α protein levels.

Increased VEGF expression through HIF-1α-independent mechanisms has been reported. For example, the RAS oncogene has been shown to be able to induce VEGF expression in human cancer cells where HIF-1α is unable to bind to the VEGF promoter due to mutations in the HIF-1α binding sites of the promoter [34,56]. NF-κB has also been shown to regulate VEGF expression in response to cytokines such as interleukin 8 (IL-8), independent of HIF-1α [34]. VEGF is induced in response to TGF-β, regardless of hypoxia [57,58]. Breast tumor cells, including MDA-MB-231 and MCF7 cells, express TGF-β and various cytokines such as IL-8. Further experiments are required to determine the mechanism by which BM-MSCs increase VEGF expression independent of HIF-1α in response to breast tumor cells. BM-MSCs, in response to breast tumor-mimicking conditions, showed increased VEGF expression in vitro under normoxic conditions. However, hypoxia is a characteristic of the tumor microenvironment [59]. Therefore, the mechanisms that mediate the increase in HIF-1α levels in BM-MSCs under hypoxic conditions mimicking the tumor microenvironment, along with the mechanisms by which BM-MSCs regulate VEGF expression, must be investigated.

## 5. Conclusions

BM-MSCs play essential roles in inducing tumor tropism in the tumor microenvironment. As tumors grow, angiogenesis is required for tumor cells’ proliferation and survival. We demonstrated that ROS and JAK/Stat3 synergistically lead to an increase in HIF-1α in BM-MSCs in vitro under normoxic conditions mimicking the breast tumor microenvironment. Furthermore, HIF-1α induced by in vitro normoxic conditions mimicking the breast tumor microenvironment promoted VEGF expression in BM-MSCs which, in turn, enhanced the angiogenic sprouting capacity of HUVECs. However, HIF-1α only partially induced VEGF expression, and BM-MSCs also showed HIF-1α-independent VEGF expression. A further understanding of the mechanisms underlying HIF-1α-independent induction of VEGF may provide therapeutic strategies for controlling angiogenesis enhanced by factors of the tumor microenvironment, such as BM-MSC secretions.

## Data Availability

The data that support the findings of this study are available from the corresponding author upon reasonable request.

## Supplementary Materials

The following supporting information can be downloaded at: https://www.mdpi.com/article/10.3390/cancers14194711/s1, Figure S1: Full unedited blots of Figure 1A,D; Figure S2: Full unedited blots of Figure 2A–K; Figure S3: Protein expression levels of HIF-1α in BM-MSCs transfected with *HIF1A* siRNA; Figure S4: qRT-PCR analysis of VEGFA of MDA-MB-231, BM-MSCs, and BM-MSCs treated with conditioned media from the control (CON CM) or from the breast tumor cell line MDA-MB-231 (MDA CM).

## References

[1] Wu Y., Chen L., Scott P.G., Tredget E.E. (2007). Mesenchymal stem cells enhance wound healing through differentiation and angiogenesis. Stem Cells.

[2] Maxson S., Lopez E.A., Yoo D., Danilkovitch-Miagkova A., Leroux M.A. (2012). Concise review: Role of mesenchymal stem cells in wound repair. Stem Cells Transl. Med..

[3] Sasaki M., Abe R., Fujita Y., Ando S., Inokuma D., Shimizu H. (2008). Mesenchymal stem cells are recruited into wounded skin and contribute to wound repair by transdifferentiation into multiple skin cell type. J. Immunol..

[4] Janeczek Portalska K., Leferink A., Groen N., Fernandes H., Moroni L., van Blitterswijk C., de Boer J. (2012). Endothelial differentiation of mesenchymal stromal cells. PLoS ONE.

[5] Pittenger M.F., Discher D.E., Peault B.M., Phinney D.G., Hare J.M., Caplan A.I. (2019). Mesenchymal stem cell perspective: Cell biology to clinical progress. NPJ Regen. Med..

[6] Chen L., Tredget E.E., Wu P.Y., Wu Y. (2008). Paracrine factors of mesenchymal stem cells recruit macrophages and endothelial lineage cells and enhance wound healing. PLoS ONE.

[7] Hanahan D., Weinberg R.A. (2011). Hallmarks of cancer: The next generation. Cell.

[8] Shi Y., Du L., Lin L., Wang Y. (2017). Tumour-associated mesenchymal stem/stromal cells: Emerging therapeutic targets. Nat. Rev. Drug Discov..

[9] Karnoub A.E., Dash A.B., Vo A.P., Sullivan A., Brooks M.W., Bell G.W., Richardson A.L., Polyak K., Tubo R., Weinberg R.A. (2007). Mesenchymal stem cells within tumour stroma promote breast cancer metastasis. Nature.

[10] Suzuki K., Sun R., Origuchi M., Kanehira M., Takahata T., Itoh J., Umezawa A., Kijima H., Fukuda S., Saijo Y. (2011). Mesenchymal stromal cells promote tumor growth through the enhancement of neovascularization. Mol. Med..

[11] Spaeth E.L., Dembinski J.L., Sasser A.K., Watson K., Klopp A., Hall B., Andreeff M., Marini F. (2009). Mesenchymal stem cell transition to tumor-associated fibroblasts contributes to fibrovascular network expansion and tumor progression. PLoS ONE.

[12] Batlle R., Andres E., Gonzalez L., Llonch E., Igea A., Gutierrez-Prat N., Berenguer-Llergo A., Nebreda A.R. (2019). Regulation of tumor angiogenesis and mesenchymal-endothelial transition by p38alpha through TGF-beta and JNK signaling. Nat. Commun..

[13] Bexell D., Gunnarsson S., Tormin A., Darabi A., Gisselsson D., Roybon L., Scheding S., Bengzon J. (2009). Bone marrow multipotent mesenchymal stroma cells act as pericyte-like migratory vehicles in experimental gliomas. Mol. Ther..

[14] Huang W.H., Chang M.C., Tsai K.S., Hung M.C., Chen H.L., Hung S.C. (2013). Mesenchymal stem cells promote growth and angiogenesis of tumors in mice. Oncogene.

[15] Orimo A., Gupta P.B., Sgroi D.C., Arenzana-Seisdedos F., Delaunay T., Naeem R., Carey V.J., Richardson A.L., Weinberg R.A. (2005). Stromal fibroblasts present in invasive human breast carcinomas promote tumor growth and angiogenesis through elevated SDF-1/CXCL12 secretion. Cell.

[16] Beckermann B.M., Kallifatidis G., Groth A., Frommhold D., Apel A., Mattern J., Salnikov A.V., Moldenhauer G., Wagner W., Diehlmann A. (2008). VEGF expression by mesenchymal stem cells contributes to angiogenesis in pancreatic carcinoma. Br. J. Cancer.

[17] Liu Y., Han Z.P., Zhang S.S., Jing Y.Y., Bu X.X., Wang C.Y., Sun K., Jiang G.C., Zhao X., Li R. (2011). Effects of inflammatory factors on mesenchymal stem cells and their role in the promotion of tumor angiogenesis in colon cancer. J. Biol. Chem..

[18] Forsythe J.A., Jiang B.H., Iyer N.V., Agani F., Leung S.W., Koos R.D., Semenza G.L. (1996). Activation of vascular endothelial growth factor gene transcription by hypoxia-inducible factor 1. Mol. Cell. Biol..

[19] Pugh C.W., Ratcliffe P.J. (2003). Regulation of angiogenesis by hypoxia: Role of the HIF system. Nat. Med..

[20] Kaelin W.G. (2005). Proline hydroxylation and gene expression. Annu. Rev. Biochem..

[21] Ivan M., Kondo K., Yang H., Kim W., Valiando J., Ohh M., Salic A., Asara J.M., Lane W.S., Kaelin W.G. (2001). HIFalpha targeted for VHL-mediated destruction by proline hydroxylation: Implications for O_2_ sensing. Science.

[22] Schofield C.J., Ratcliffe P.J. (2004). Oxygen sensing by HIF hydroxylases. Nat. Rev. Mol. Cell Biol..

[23] Iommarini L., Porcelli A.M., Gasparre G., Kurelac I. (2017). Non-Canonical Mechanisms Regulating Hypoxia-Inducible Factor 1 Alpha in Cancer. Front. Oncol..

[24] Sonveaux P., Copetti T., De Saedeleer C.J., Vegran F., Verrax J., Kennedy K.M., Moon E.J., Dhup S., Danhier P., Frerart F. (2012). Targeting the lactate transporter MCT1 in endothelial cells inhibits lactate-induced HIF-1 activation and tumor angiogenesis. PLoS ONE.

[25] Lu H., Forbes R.A., Verma A. (2002). Hypoxia-inducible factor 1 activation by aerobic glycolysis implicates the Warburg effect in carcinogenesis. J. Biol. Chem..

[26] Dewhirst M.W., Cao Y., Moeller B. (2008). Cycling hypoxia and free radicals regulate angiogenesis and radiotherapy response. Nat. Rev. Cancer.

[27] Kietzmann T., Mennerich D., Dimova E.Y. (2016). Hypoxia-Inducible Factors (HIFs) and Phosphorylation: Impact on Stability, Localization, and Transactivity. Front. Cell Dev. Biol..

[28] Xu Q., Briggs J., Park S., Niu G., Kortylewski M., Zhang S., Gritsko T., Turkson J., Kay H., Semenza G.L. (2005). Targeting Stat3 blocks both HIF-1 and VEGF expression induced by multiple oncogenic growth signaling pathways. Oncogene.

[29] Arany Z., Foo S.Y., Ma Y., Ruas J.L., Bommi-Reddy A., Girnun G., Cooper M., Laznik D., Chinsomboon J., Rangwala S.M. (2008). HIF-independent regulation of VEGF and angiogenesis by the transcriptional coactivator PGC-1alpha. Nature.

[30] Shi Q., Le X., Wang B., Abbruzzese J.L., Xiong Q., He Y., Xie K. (2001). Regulation of vascular endothelial growth factor expression by acidosis in human cancer cells. Oncogene.

[31] Fukumura D., Xu L., Chen Y., Gohongi T., Seed B., Jain R.K. (2001). Hypoxia and acidosis independently up-regulate vascular endothelial growth factor transcription in brain tumors in vivo. Cancer Res..

[32] Sodhi A., Montaner S., Miyazaki H., Gutkind J.S. (2001). MAPK and Akt act cooperatively but independently on hypoxia inducible factor-1alpha in rasV12 upregulation of VEGF. Biochem. Biophys. Res. Commun..

[33] Richard D.E., Berra E., Pouyssegur J. (2000). Nonhypoxic pathway mediates the induction of hypoxia-inducible factor 1alpha in vascular smooth muscle cells. J. Biol. Chem..

[34] Mizukami Y., Kohgo Y., Chung D.C. (2007). Hypoxia inducible factor-1 independent pathways in tumor angiogenesis. Clin. Cancer Res..

[35] Wei D., Le X., Zheng L., Wang L., Frey J.A., Gao A.C., Peng Z., Huang S., Xiong H.Q., Abbruzzese J.L. (2003). Stat3 activation regulates the expression of vascular endothelial growth factor and human pancreatic cancer angiogenesis and metastasis. Oncogene.

[36] Jung J.E., Kim H.S., Lee C.S., Shin Y.J., Kim Y.N., Kang G.H., Kim T.Y., Juhnn Y.S., Kim S.J., Park J.W. (2008). STAT3 inhibits the degradation of HIF-1alpha by pVHL-mediated ubiquitination. Exp. Mol. Med..

[37] Fang L., Li Y., Wang S., Li Y., Chang H.M., Yi Y., Yan Y., Thakur A., Leung P.C.K., Cheng J.C. (2020). TGF-beta1 induces VEGF expression in human granulosa-lutein cells: A potential mechanism for the pathogenesis of ovarian hyperstimulation syndrome. Exp. Mol. Med..

[38] Leonard W.J., O’Shea J.J. (1998). Jaks and STATs: Biological implications. Annu. Rev. Immunol..

[39] Villarino A.V., Kanno Y., O’Shea J.J. (2017). Mechanisms and consequences of Jak-STAT signaling in the immune system. Nat. Immunol..

[40] Uemura R., Xu M., Ahmad N., Ashraf M. (2006). Bone marrow stem cells prevent left ventricular remodeling of ischemic heart through paracrine signaling. Circ. Res..

[41] Yang K.Q., Liu Y., Huang Q.H., Mo N., Zhang Q.Y., Meng Q.G., Cheng J.W. (2017). Bone marrow-derived mesenchymal stem cells induced by inflammatory cytokines produce angiogenetic factors and promote prostate cancer growth. BMC Cancer.

[42] Zhang T., Lee Y.W., Rui Y.F., Cheng T.Y., Jiang X.H., Li G. (2013). Bone marrow-derived mesenchymal stem cells promote growth and angiogenesis of breast and prostate tumors. Stem Cell Res. Ther..

[43] Choi S., Yu J., Kim W., Park K.S. (2021). N-cadherin mediates the migration of bone marrow-derived mesenchymal stem cells toward breast tumor cells. Theranostics.

[44] Bournazou E., Bromberg J. (2013). Targeting the tumor microenvironment: JAK-STAT3 signaling. JAKSTAT.

[45] Zou S., Tong Q., Liu B., Huang W., Tian Y., Fu X. (2020). Targeting STAT3 in Cancer Immunotherapy. Mol. Cancer.

[46] Jung J.E., Lee H.G., Cho I.H., Chung D.H., Yoon S.H., Yang Y.M., Lee J.W., Choi S., Park J.W., Ye S.K. (2005). STAT3 is a potential modulator of HIF-1-mediated VEGF expression in human renal carcinoma cells. FASEB J..

[47] La Rosee F., Bremer H.C., Gehrke I., Kehr A., Hochhaus A., Birndt S., Fellhauer M., Henkes M., Kumle B., Russo S.G. (2020). The Janus kinase 1/2 inhibitor ruxolitinib in COVID-19 with severe systemic hyperinflammation. Leukemia.

[48] Simon A.R., Rai U., Fanburg B.L., Cochran B.H. (1998). Activation of the JAK-STAT pathway by reactive oxygen species. Am. J. Physiol..

[49] Pan Y., Mansfield K.D., Bertozzi C.C., Rudenko V., Chan D.A., Giaccia A.J., Simon M.C. (2007). Multiple factors affecting cellular redox status and energy metabolism modulate hypoxia-inducible factor prolyl hydroxylase activity in vivo and in vitro. Mol. Cell. Biol..

[50] Chandel N.S., Maltepe E., Goldwasser E., Mathieu C.E., Simon M.C., Schumacker P.T. (1998). Mitochondrial reactive oxygen species trigger hypoxia-induced transcription. Proc. Natl. Acad. Sci. USA.

[51] Bonello S., Zahringer C., BelAiba R.S., Djordjevic T., Hess J., Michiels C., Kietzmann T., Gorlach A. (2007). Reactive oxygen species activate the HIF-1alpha promoter via a functional NFkappaB site. Arterioscler. Thromb. Vasc. Biol..

[52] Choi S., Yu J., Park A., Dubon M.J., Do J., Kim Y., Nam D., Noh J., Park K.S. (2019). BMP-4 enhances epithelial mesenchymal transition and cancer stem cell properties of breast cancer cells via Notch signaling. Sci. Rep..

[53] Wilhelm S.M., Carter C., Tang L., Wilkie D., McNabola A., Rong H., Chen C., Zhang X., Vincent P., McHugh M. (2004). BAY 43-9006 exhibits broad spectrum oral antitumor activity and targets the RAF/MEK/ERK pathway and receptor tyrosine kinases involved in tumor progression and angiogenesis. Cancer Res..

[54] Wilhelm S.M., Adnane L., Newell P., Villanueva A., Llovet J.M., Lynch M. (2008). Preclinical overview of sorafenib, a multikinase inhibitor that targets both Raf and VEGF and PDGF receptor tyrosine kinase signaling. Mol. Cancer Ther..

[55] Lacher S.E., Levings D.C., Freeman S., Slattery M. (2018). Identification of a functional antioxidant response element at the *HIF1A* locus. Redox Biol..

[56] Mizukami Y., Li J., Zhang X., Zimmer M.A., Iliopoulos O., Chung D.C. (2004). Hypoxia-inducible factor-1-independent regulation of vascular endothelial growth factor by hypoxia in colon cancer. Cancer Res..

[57] Nakagawa T., Lan H.Y., Zhu H.J., Kang D.H., Schreiner G.F., Johnson R.J. (2004). Differential regulation of VEGF by TGF-beta and hypoxia in rat proximal tubular cells. Am. J. Physiol.-Ren. Physiol..

[58] Jeon S.H., Chae B.C., Kim H.A., Seo G.Y., Seo D.W., Chun G.T., Kim N.S., Yie S.W., Byeon W.H., Eom S.H. (2007). Mechanisms underlying TGF-beta1-induced expression of VEGF and Flk-1 in mouse macrophages and their implications for angiogenesis. J. Leukoc. Biol..

[59] Vaupel P., Hockel M., Mayer A. (2007). Detection and characterization of tumor hypoxia using pO2 histography. Antioxid. Redox Signal..

