# Supplementary material for: Breast Tumor Cell-Stimulated Bone Marrow-Derived Mesenchymal Stem Cells Promote the Sprouting Capacity of Endothelial Cells by Promoting VEGF Expression, Mediated in Part through HIF-1α Increase
Source: Cancers (Basel). 2022 Sep 27;14(19):4711. doi: 10.3390/cancers14194711 (PMC9562024; doi:10.3390/cancers14194711)
Supplement: Supplementary file 1 [file cancers-14-04711-s001.zip › cancers-1905594-supplementary.pdf]

Breast Tumor Cell-Stimulated Bone Marrow-Derived Mesenchymal Stem Cells Promote the Sprouting Capacity of Endothelial Cells by Promoting VEGF Expression, Mediated in Part through HIF-1α Increase

Wootak Kim [1,†], Aran Park [2,†], Hyun Hee Jang [2], Seung-Eun Kim [3] and Ki-Sook Park [1,4,*]

1 Department of Biomedical Science and Technology, Graduate School, Kyung Hee University, Seoul 02447, Korea
2 Graduate School of Biotechnology, Kyung Hee University, Yongin 17104, Korea
3 Department of Genetics and Biotechnology, Kyung Hee University, Yongin 17104, Korea
4 East-West Medical Research Institute, Kyung Hee University, Seoul 02447, Korea
* Correspondence: kisookpark@khu.ac.kr; Tel.: +82-2-958-9368
† These authors contributed equally to this work.

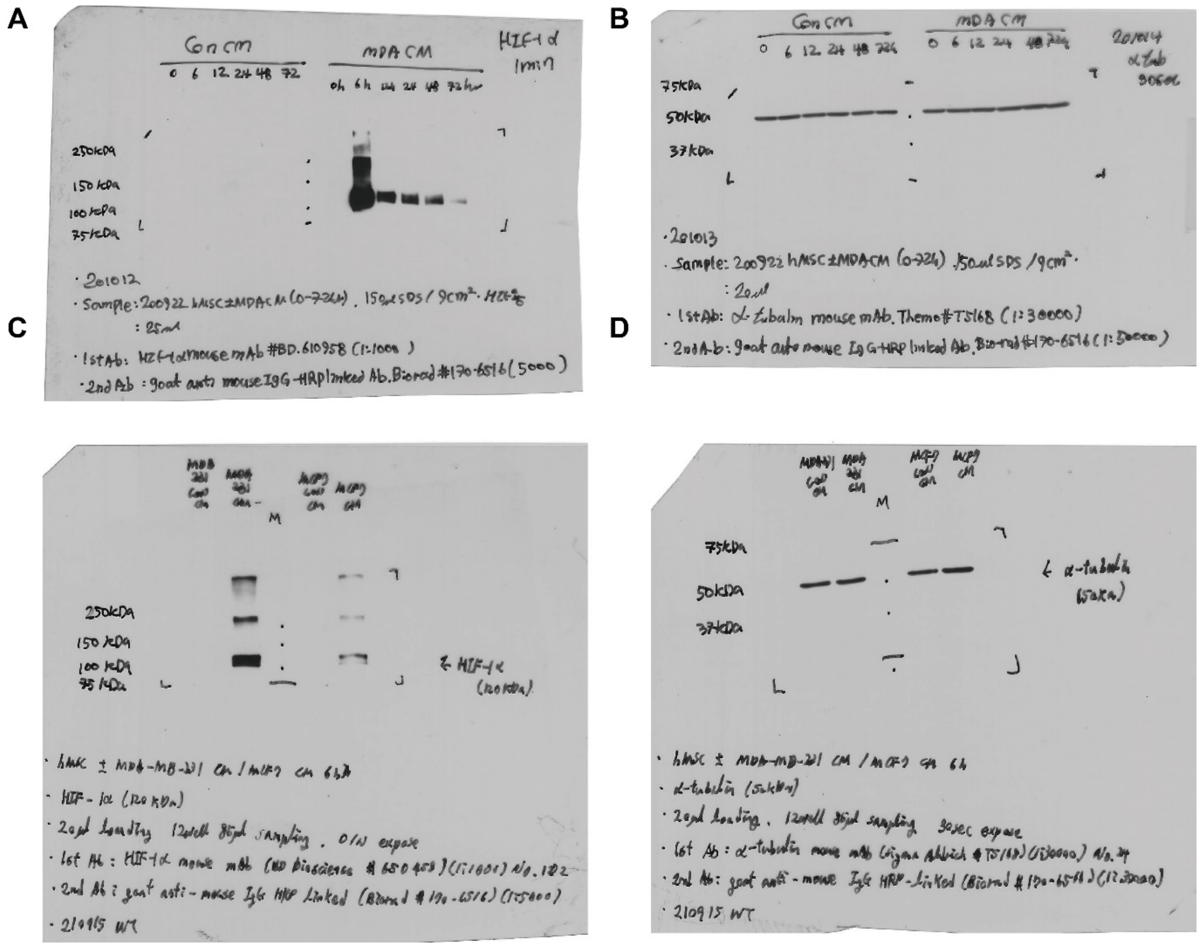

Figure S1. Full unedited blots of Figure 1A and 1D. (**A**, **B**) Full unedited blots for Figure 1A; HIF-1α and α-tubulin. (**C**, **D**) Full unedited blots for Figure 1D; HIF-1α and α-tubulin.

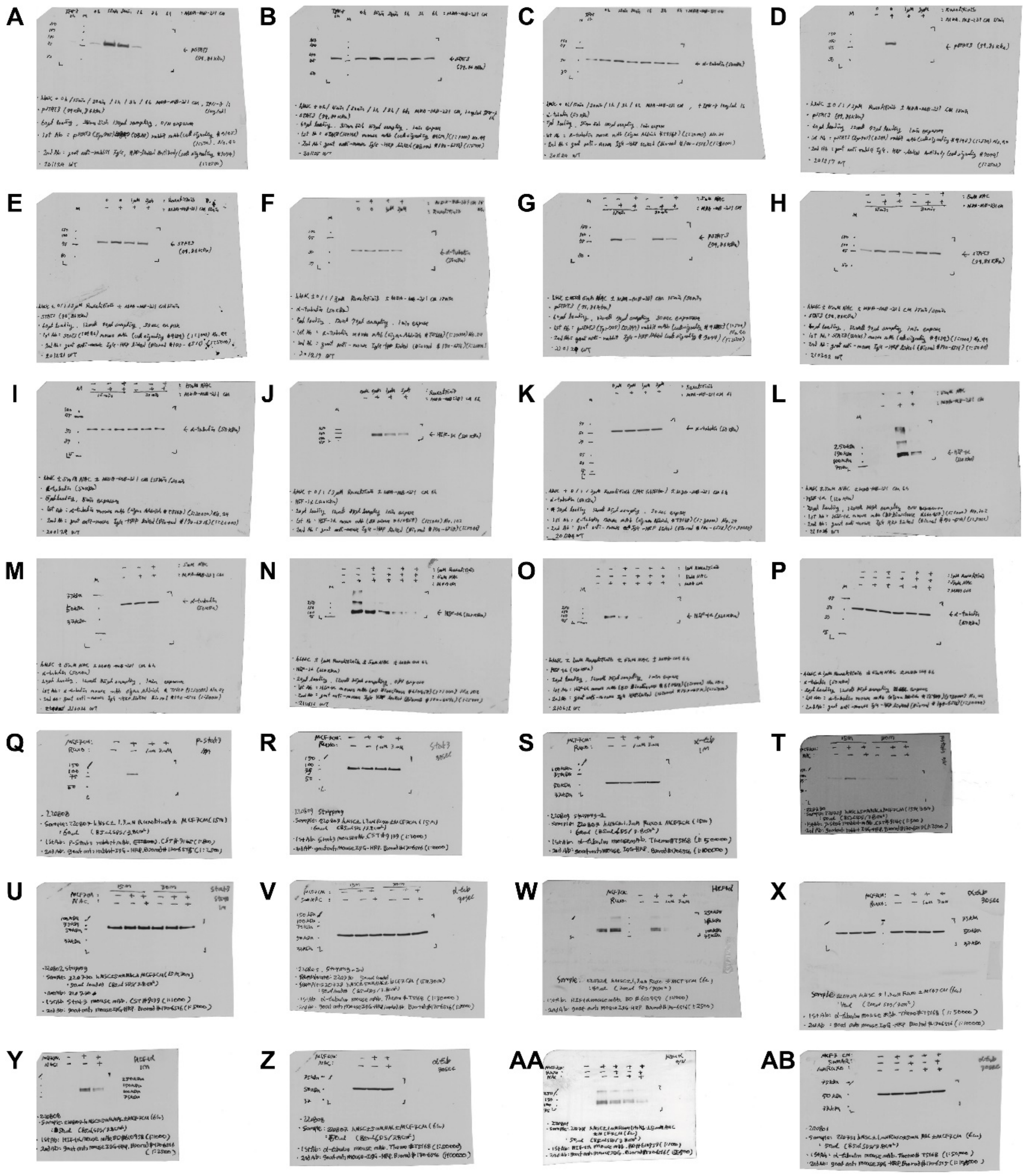

Figure S2. Full unedited blots of Figure 2A-2K. (**A**--**C**) Full unedited blots for Figure 2A; pStat3, Stat3, and α-tubulin, respectively. (**D**--**F**) Full unedited blots for Figure 2B; pStat3, Stat3, and α-tubulin, respectively. (**G**--**I**) Full unedited blots for Figure 2C; pStat3, Stat3, and α-tubulin, respectively. (**J**, **K**) Full unedited blots for Figure 2D; HIF-1α and α-tubulin. (**L**,**M**) Full unedited blots for Figure 2E; HIF-1α and α-tubulin. (**N**--**P**) Full unedited blots for Figure 2F; HIF-1α, HIF-1α, and α-tubulin, respectively. (**Q**--**S**) Full unedited blots for Figure 2G; pStat3, Stat3, and α-tubulin, respectively. (**T**--**V**) Full unedited blots for Figure 2H; pStat3, Stat3, and α-tubulin, respectively. (**W**, **X**) Full unedited blots for Figure 2I; HIF-1α and α-tubulin. (**Y**, **Z**) Full unedited blots for Figure 2J; HIF-1α and α-tubulin. (**AA**, **AB**) Full unedited blots for Figure 2K; HIF-1α and α-tubulin.

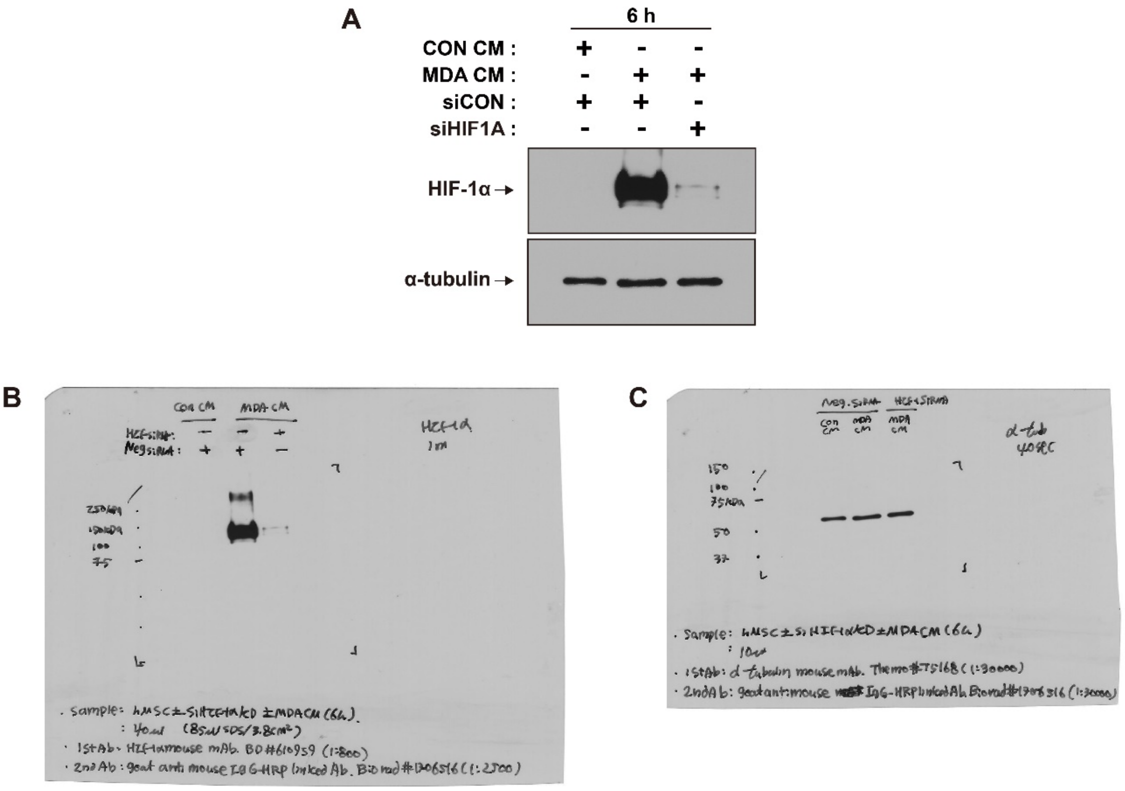

Figure S3. Protein expression level of HIF-1α in BM-MSCs transfected with HIF1A siRNA. (**A**) Western blot analysis HIF-1α and α-tubulin in BM-MSCs transfected with HIF1A siRNA or control siRNA prior to treatment with CON CM or MDA CM for 6 h. (**B**,**C**) Full unedited blots of panel A; HIF-1α and α-tubulin, respectively.

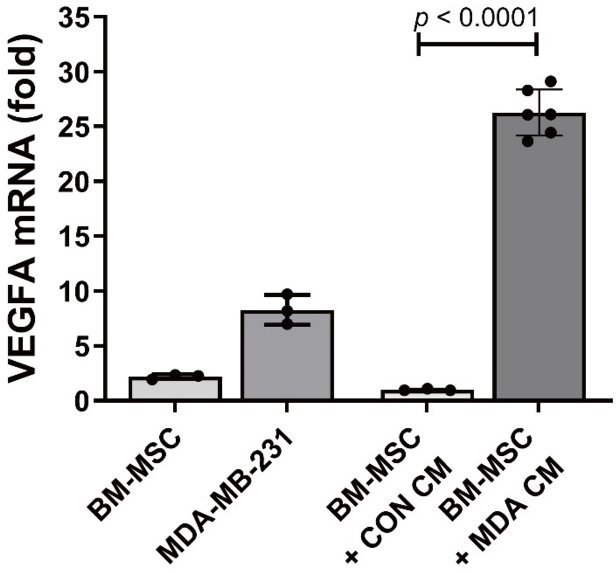

Figure S4. qRT-PCR analysis of VEGFA of MDA-MB-231, BM-MSCs, and BM-MSCs treated with conditioned media from the control (CON CM) or from the breast tumor cell line MDA-MB-231 (MDA CM). Results are presented as the mean ± SD.
